# Quantifying post-treatment vascular remodeling in brain aneurysms using WEKA-based machine learning: a pilot study

**DOI:** 10.3389/fneur.2025.1650932

**Published:** 2025-10-09

**Authors:** Ante Rotim, Marina Raguž, Nikica Fulir, Darko Orešković, Vladimir Kalousek, Petar Marčinković, Krešimir Rotim, Bruno Splavski, Silva Butković Soldo, Tomislav Sajko

**Affiliations:** ^1^Department of Neurosurgery, Sestre Milosrdnice University Hospital Center, Zagreb, Croatia; ^2^Faculty of Medicine, Josip Juraj Strossmayer University of Osijek, Osijek, Croatia; ^3^Department of Neurosurgery, Dubrava University Hospital, Zagreb, Croatia; ^4^School of Medicine, Catholic University of Croatia, Zagreb, Croatia; ^5^Department of Radiology, Sestre Milosrdnice University Hospital Center, Zagreb, Croatia; ^6^Special Hospital Neurospine, Zagreb, Croatia; ^7^University of Applied Health Sciences, Zagreb, Croatia; ^8^Department of Neurosurgery, Dubrovnik General Hospital, Dubrovnik, Croatia; ^9^Department of Neurology, University Hospital Center Osijek, Osijek, Croatia

**Keywords:** intracranial aneurysms, middle cerebral artery, WEKA-based segmentation, machine learning, aneurysm treatment outcome, hemodynamic remodeling

## Abstract

**Introduction:**

To evaluate the feasibility of a WEKA-based machine learning pipeline for detecting post-treatment hemodynamic remodeling by comparing pre- and postoperative cerebral angiographic images in patients with middle cerebral artery aneurysms.

**Methods:**

This retrospective, single-center study analyzed 60 patients (51 women, 9 men; mean age, 58.2 ± 10.2 years) with unruptured middle cerebral artery aneurysms treated between January 2019 and June 2024. Thirty patients underwent microsurgical clipping, and 29 underwent endovascular intervention. A WEKA-based Random Forest classifier was trained on 15 manually annotated pre- and postoperative digital subtraction angiography (DSA) image pairs and then applied to the remaining dataset. Custom Python-based post-processing was used to denoise and refine the segmented images. Vascular surface area changes were assessed by comparing pixel counts before and after treatment. Statistical analysis included paired and unpaired t-tests, Mann-Whitney U tests, and effect size estimation.

**Results:**

Among 51 analyzable image pairs, 75% showed increased vascular pixel counts postoperatively, particularly in the endovascular group (segmented pixels: *p* = 0.034; refined pixels: *p* = 0.017). No statistically significant differences were observed in the neurosurgical group. Between-group comparisons of postoperative images did not reach significance.

**Conclusion:**

The WEKA pipeline enabled quantification of vascular remodeling but remained limited by manual preprocessing and lack of external validation. Machine learning–guided segmentation of angiographic images can detect treatment-induced vascular changes, particularly following endovascular therapy. This method demonstrates promise for future development of automated imaging biomarkers to support outcome monitoring and clinical decision-making in neurovascular care.

## Introduction

The successful treatment of brain aneurysms requires a multidisciplinary approach, integrating advanced imaging modalities with clinical expertise. Digital subtraction angiography (DSA) remains the gold standard in both preoperative planning and postoperative monitoring due to its unmatched resolution and ability to assess vascular anatomy and aneurysm occlusion (1, 2) accurately. It enables precise localization and morphological analysis of aneurysms, guiding optimal therapeutic strategies (3, 4), and serves as a critical tool in confirming treatment success, with complete angiographic occlusion significantly reducing recurrence risk (5, 6). Despite improvements in microsurgical and endovascular techniques, postoperative vascular remodeling remains a dynamic process that is best evaluated through high-resolution imaging. Post-treatment DSA, complemented by CT or MR angiography, is essential for detecting residual or recurrent aneurysms, vessel narrowing, or delayed complications (7, 8). However, inconsistent follow-up imaging, particularly in surgically treated patients, can result in missed detections of critical changes such as aneurysm remnants, *de novo* aneurysm formation, pseudoaneurysms, or procedural complications (8–11). DSA also provides unparalleled insight into dynamic vascular processes such as vasospasm and thrombosis, especially after subarachnoid hemorrhage (11, 12). When performed both pre- and postoperatively, DSA enables objective assessment of vascular changes over time and supports outcome tracking for clinical decision-making and research.

Technological advances such as 3D rotational angiography have further improved spatial resolution and vascular visualization (13). At the same time, artificial intelligence (AI) and machine learning (ML) are transforming neuroimaging by automating complex tasks, increasing detection accuracy, and facilitating large-scale image analysis. Deep learning models, particularly convolutional neural networks (CNNs), have demonstrated strong performance in neurovascular segmentation tasks (14, 15). Within this evolving landscape, the WEKA platform offers a versatile and accessible tool for machine learning–based image segmentation. Specifically, WEKA is leveraged for pixel-wise vascular segmentation of DSA images, enabling quantitative assessment of blood vessel surface area and its modifications across treatment stages. We hypothesized that WEKA-based machine learning segmentation could quantitatively detect vascular changes between pre- and postoperative angiographic images, particularly in the endovascular cohort, due to its greater propensity for hemodynamic remodeling. In this study, vascular remodeling is defined as image-based morphological change, specifically, variation in segmented vessel surface area between pre- and postoperative angiographic images, which may indirectly reflect hemodynamic adaptation following treatment.

Previous studies have documented post-treatment changes in vessel morphology, including diameter, angle, and curvature, that are interpreted as indirect evidence of vascular remodeling, particularly after stenting or coiling procedures (16, 17). Building on this foundation, the present study applies a WEKA-based segmentation approach to evaluate postoperative hemodynamic remodeling in patients with middle cerebral artery aneurysms treated by clipping or endovascular intervention. By quantifying pixel-level changes between pre- and postoperative images, this method provides an objective, data-driven measure of vascular response to treatment. While sample size and manual preprocessing limit this preliminary analysis, it lays the groundwork for future development of fully automated, AI-guided imaging pipelines and highlights the clinical potential of machine learning in enhancing precision and reproducibility in neurovascular outcome evaluation.

## Methods

**Figure 1:**
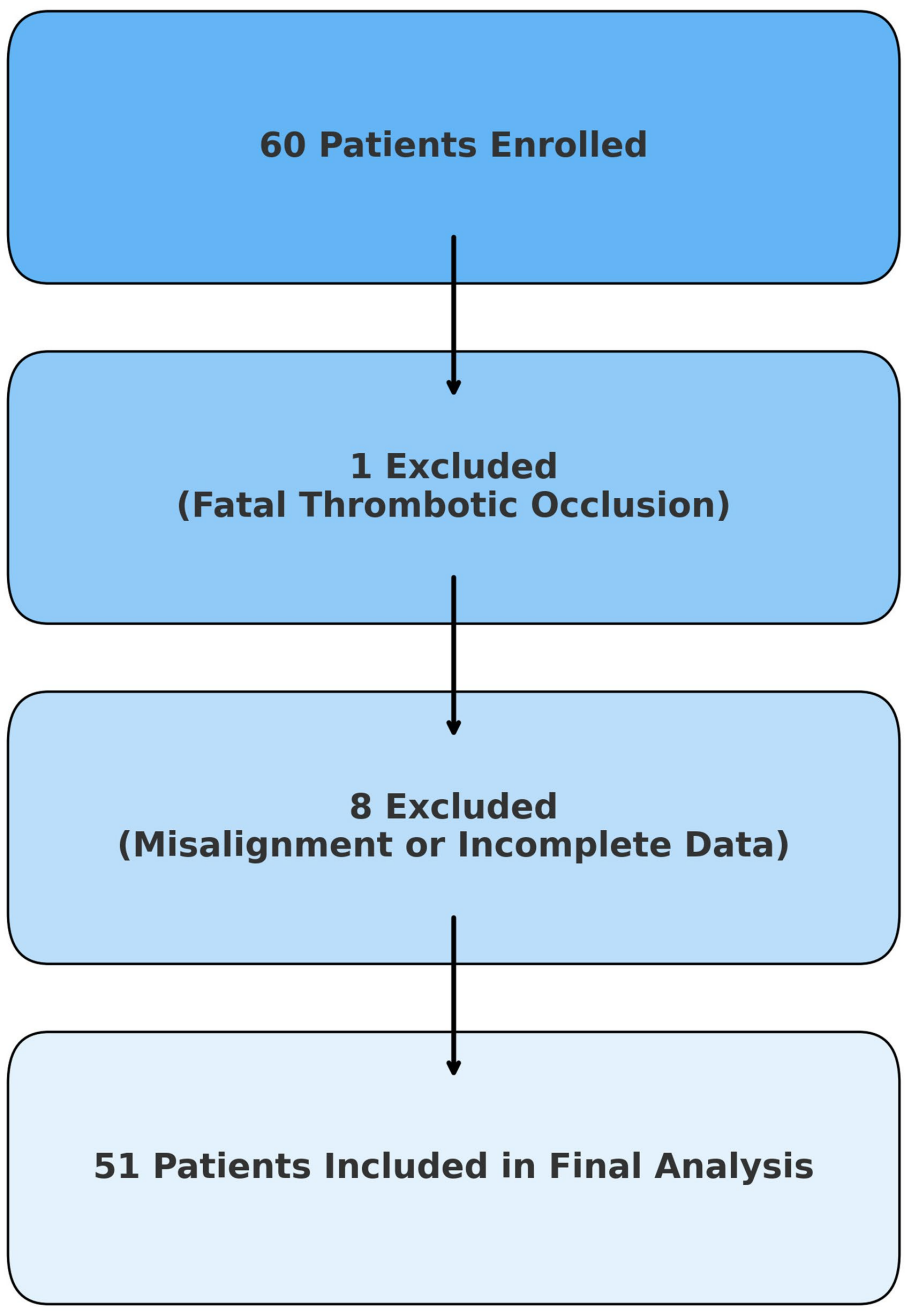
Flowchart of patient inclusion and exclusion criteria for final analysis. This figure illustrates the inclusion and exclusion process for the final patient cohort. Of 60 initially enrolled patients, 1 was excluded due to a fatal thrombotic complication, and 8 were excluded due to severe image misalignment or incomplete data, resulting in 51 image pairs eligible for final analysis.

This retrospective, single-center study included 60 consecutive adult patients diagnosed with unruptured middle cerebral artery (MCA) aneurysms. We limited the analysis to MCA aneurysms to minimize anatomical and imaging variability and to ensure uniform projection angles during acquisition. All patients were treated at the Department of Neurosurgery and the Clinical Department of Diagnostic and Interventional Radiology, University Hospital Center Sestre Milosrdnice, Zagreb, Croatia, between January 1, 2019, and June 30, 2024. Treatment decisions were made by a multidisciplinary team consisting of neurosurgeons, neurologists, and radiologists. Patients were thoroughly informed about the risks and benefits of both treatment options before providing written informed consent. Based on the selected treatment modality, patients were divided into two cohorts. The neurosurgical group consisted of 30 patients (27 women, 3 men; mean age, 57.5 ± 11.4 years) who were treated with microsurgical clipping. The endovascular group initially consisted of 30 patients; however, one was excluded due to non-compliance with dual antiplatelet therapy, resulting in fatal thrombotic occlusion of a flow diverter. The final endovascular cohort consisted of 29 patients (24 women, 5 men; mean age, 59.0 ± 9.0 years). Additionally, eight cases were excluded from the final analysis due to technical limitations that prevented reliable image segmentation. These included cases with severe pre- or postoperative image misalignment, missing follow-up angiograms, or low-quality contrast enhancement. Exclusions were not related to treatment outcomes and were applied uniformly based on objective imaging criteria. The patient selection and exclusion process is summarized in Figure 1.

Exclusion criteria were ruptured aneurysms, anatomical variants precluding accurate assessment (e.g., severe vessel tortuosity, distal stenosis), non-compliance with prescribed therapy, and lack of postoperative DSA follow-up. All DSA procedures—both preoperative (1–3 days before intervention) and postoperative (6–12 months after treatment)—were performed using the same biplane angiographic system (Siemens Artis zee, Germany) with standardized acquisition parameters. Imaging protocols were strictly matched across timepoints to eliminate technical variability and ensure consistent pixel-based analysis. This included consistent patient positioning, identical contrast agent dose and injection timing, and use of the same biplane DSA system for all acquisitions. These measures were taken to minimize variability in vascular contrast enhancement and geometric projection, thereby enhancing the comparability of segmented images across timepoints. Operator consistency was maintained by having all procedures performed by the same neurosurgeon or interventional neuroradiologist.

The study received institutional ethics approval and was conducted following the Declaration of Helsinki. Written informed consent was obtained from all participants (2021/602-04/21-08/07).

### Baseline aneurysm and clinical characteristics

Baseline morphometric analysis revealed that patients in the neurosurgical group had larger aneurysms on average compared to those in the endovascular group. Specifically, the mean neck width was 3.01 ± 0.61 mm and mean fundus size was 8.03 ± 2.01 mm in the neurosurgical group, versus 2.85 ± 0.59 mm and 5.13 ± 1.73 in the endovascular group, respectively. Furthermore, 45% of patients in the neurosurgical group had aneurysms larger than 7 mm, compared to 27% in the endovascular group. All patients presented with a preoperative Glasgow Coma Scale score of 15. There were no major intraoperative or postoperative complications. Functional recovery was comparable across groups, with all patients achieving a Glasgow Outcome Scale - Extended score of 8 at follow-up.

### WEKA-based image segmentation and post-processing

**Figure 2:**
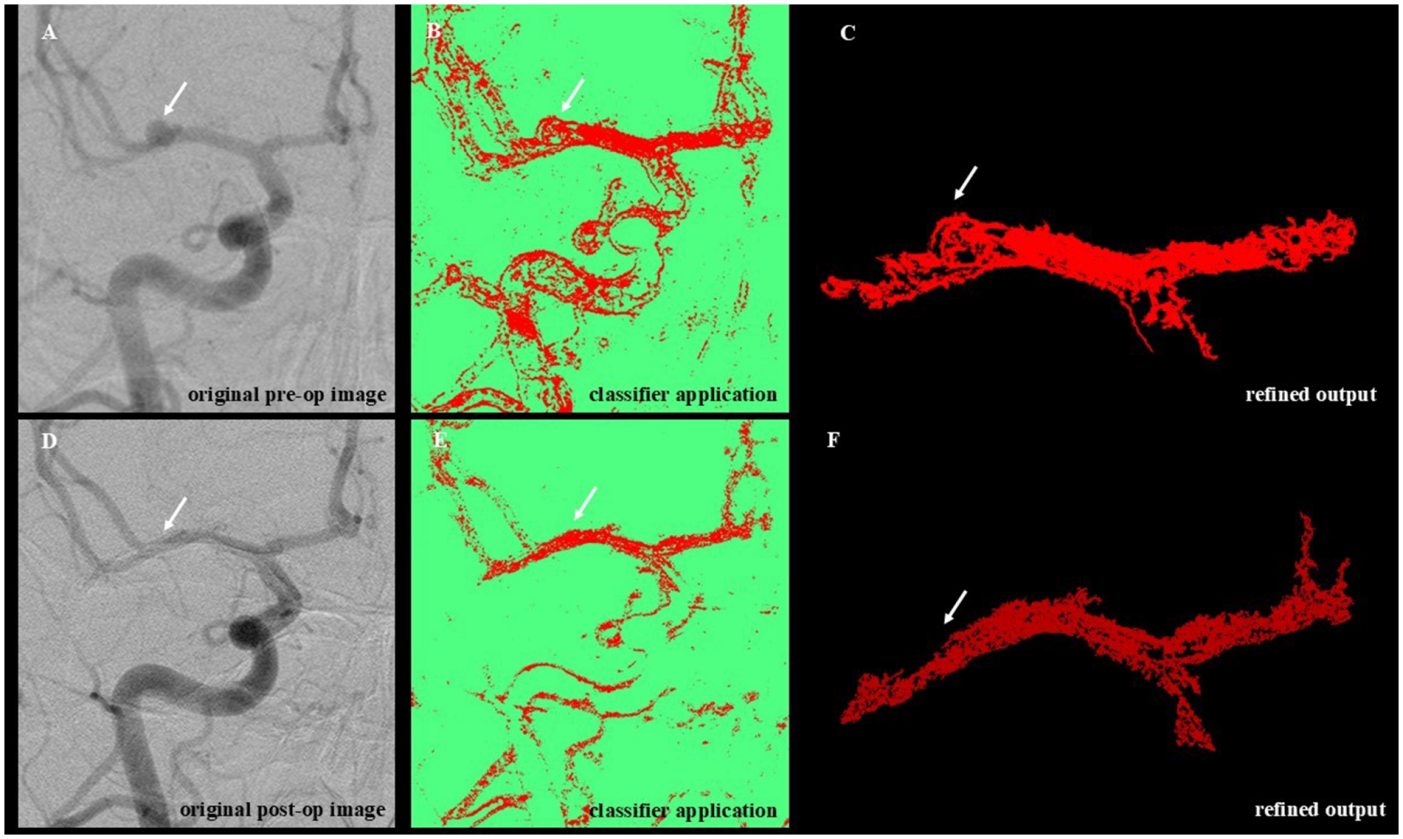
Workflow for WEKA-Based segmentation and denoising of angiographic images. This figure illustrates the sequential steps of the image analysis pipeline applied to preoperative and postoperative DSA images. **(A)** Original preoperative image showing unprocessed vascular anatomy with visible aneurysmal dilation (white arrow). **(B)** Machine learning–based segmentation using the trained WEKA classifier highlights vascular structures and the aneurysmal region. **(C)** Post-processing of the segmented image (denoising) removes background artifacts and isolates the target vascular segment. **(D)** Original postoperative angiographic image acquired following aneurysm treatment. **(E)** Application of the trained classifier to the postoperative image, enabling direct comparison with the preoperative segmentation. **(F)** Refined output after denoising, showing the treated vessel segment. The aneurysmal dilation visible preoperatively (top row) is absent in the postoperative segmentation (bottom row), indicating successful obliteration.

To assess vascular changes, we applied a WEKA-based machine learning pipeline to pre- and postoperative angiographic images. The objective was to quantify treatment-induced remodeling by calculating differences in segmented vessel area. The analytic workflow is illustrated in Figure 2. WEKA (Waikato Environment for Knowledge Analysis, version 3.9.6., University of Waikato, New Zealand) is an open-source machine-learning platform with a graphical interface that supports supervised learning techniques, including classification and regression (18). It was accessed through the Trainable WEKA Segmentation (TWS) plugin integrated into Fiji/ImageJ (19, 20), a widely used biomedical image analysis suite (21).

The classification pipeline involved a two-step process: training and application. During training, the Random Forest algorithm (22, 23) was iteratively taught to distinguish vessel structures (Class 1) from background (Class 2) using 15 angiographic image pairs. These images were manually annotated and selected for their variability, ensuring model generalizability. Visual validation was conducted at each iteration to refine accuracy. The resulting classifier, exceeding 100 MB in size, was optimized for batch analysis. Standardized preprocessing was performed using Fiji/ImageJ and included cropping, rotation alignment, contrast normalization, and resizing. These steps addressed technical variation in image orientation, head positioning, and acquisition field, minimizing artifacts due to non-biological factors such as differing injection contrast timing or device settings. The 15 pre- and postoperative image pairs used for classifier training were manually annotated using pixel-level segmentation in Fiji (ImageJ), referencing visible angiographic vessel boundaries. The WEKA classifier was trained using the FastRandomForest algorithm with default parameters. Segmentation was performed iteratively for each image pair (typically 3–5 rounds) until satisfactory vessel boundary separation was achieved. Each classifier was applied to its corresponding matched image pair (i.e., intra-subject), and cross-validation was not performed, as the goal was not to generalize across cases but to optimize pairwise segmentation fidelity. All preprocessing steps, including image rotation, cropping, and contrast adjustment, were performed in Fiji (ImageJ) to enhance vessel visualization and achieve spatial alignment between pre- and postoperative images. To minimize variability, the same reviewer processed both images in each pair using consistent parameters and anatomical landmarks. The trained classifier was applied to 53 image pairs, of which 51 were successfully segmented. Two image sets were excluded due to severe misalignment that precluded meaningful comparison. Segmentation outputs were further refined using a custom-built Python post-processing script (available upon request), leveraging the scikit-image library (24). The denoising workflow included grayscale conversion, morphological filtering, region labeling, and size-based exclusion of small artifacts. Final masks retained the largest connected vascular region to enhance specificity (Figures 2C,F). In this context, we define “refined pixels” as the total number of vascular-classified pixels remaining after post-processing, which includes noise suppression, removal of non-connected or spurious regions, and masking to preserve only the primary vascular structure. This metric reflects a cleaner and more specific measurement of vessel area than the raw segmented output, accounting for variability in contrast, acquisition artifacts, and misclassifications. Final refined pixel counts were obtained by summing all white (i.e., segmented) pixels within the middle cerebral artery region, separately for each pre- and postoperative image. These values served as a surrogate for relative vessel surface area. Although a single trained reviewer performed all image preprocessing to ensure consistency, future applications of this pipeline may require inter-rater reproducibility testing to ensure objectivity and scalability.

Across the 51 analyzable image pairs, 38 (approximately 75%) exhibited a postoperative increase in vessel pixel count, particularly among endovascularly treated patients. This pattern suggests postoperative vascular remodeling is potentially linked to altered flow dynamics. Despite variability in image quality and limited training data, the WEKA-based Random Forest classifier proved robust. To promote reproducibility and transparency, the segmentation pipeline (including the Python post-processing tool) will be made available as supplementary material upon publication. Future development should focus on deep learning models, e.g., convolutional neural networks, semi-supervised learning, and AutoML approaches, to enhance scalability and reduce manual input.

### Statistical analysis

All statistical analyses were performed using MedCalc Statistical Software (version 12.5.0, Ostend, Belgium). Continuous variables were expressed as mean ± standard deviation for normally distributed data, or as median and interquartile range (IQR) for non-normally distributed data. Normality was assessed using the Shapiro–Wilk test for all continuous variables. Since all images were acquired with consistent spatial resolution and processed using standardized preprocessing parameters, relative differences in segmented pixel counts were treated as proportional surrogates for vascular surface area changes. To compare preoperative and postoperative values within each treatment group, the Wilcoxon Signed-Rank test was used for non-normally distributed data, and paired t-tests were applied when normality was confirmed. For between-group comparisons (microsurgical vs. endovascular), the Student’s t-test was used for normally distributed variables, while the Mann–Whitney U test was applied when normality assumptions were not met or when group sizes were unequal. A *p*-value of < 0.05 was considered statistically significant.

## Results

**Figure 3:**
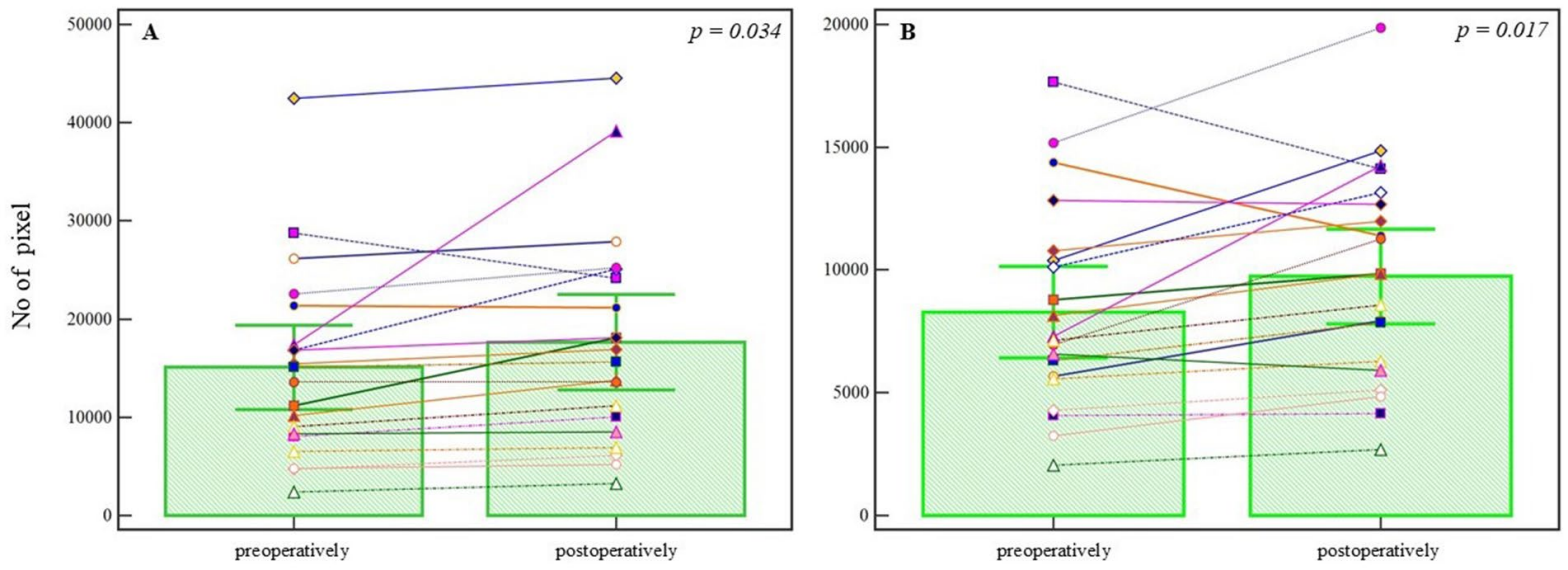
Pixel-based analysis of preoperative and postoperative angiographic images in the endovascular treatment group. **(A)** Segmented blood vessel pixels show a statistically significant increase postoperatively (*p* = 0.034, Student’s *t*-test). **(B)** Refined (denoised) pixel count also increases significantly following treatment (*p* = 0.017, Student’s *t*-test). Bars represent group means; whiskers denote ±1 standard deviation. This plot is not a traditional boxplot but illustrates mean-centered group differences and variability. *p*-values are annotated above the comparisons.

In the endovascularly treated group (*n* = 21), segmentation analysis revealed a statistically significant increase in the number of blood vessel pixels following treatment (preoperative: 15098.095 ± 9456.801 vs. postoperative: 17624.571 ± 10701.154; *T* = 2.274, DF = 20, *p* = 0.034), as shown in Figure 3A. The analysis of refined (denoised) pixels also demonstrated a statistically significant increase in postoperative images (preoperative: 8273.809 ± 4090.084 vs. postoperative: 9739.000 ± 4259.035; *T* = 2.776, DF = 20, *p* = 0.017), supporting the observation of vascular modifications following endovascular intervention (Figure 3B). Both effects corresponded to a small-to-moderate effect size (Cohen’s d = 0.35), which may hold clinical relevance in the context of subtle post-treatment vascular remodeling. A summary of within-group comparisons is presented in Table 1.

**Table 1:** Summary of pre- and postoperative segmented and refined pixel counts for endovascular and neurosurgical treatment groups, including *p*-values.

Treatment group	Preoperative segmented pixels (Mean ± SD)	Postoperative segmented pixels (Mean ± SD)	*p*-value (segmented)	Preoperative refined pixels (Mean ± SD)	Postoperative refined pixels (Mean ± SD)	*p*-value (refined)
Endovascular	15.098 ± 9.457	17.625 ± 10.701	0.034	8.274 ± 4.090	9.739 ± 4.259	0.017
Neurosurgical	13.271 (median)	13.352 (median)	0.929	6.398 (median)	6.368 (median)	0.745

**Figure 4:**
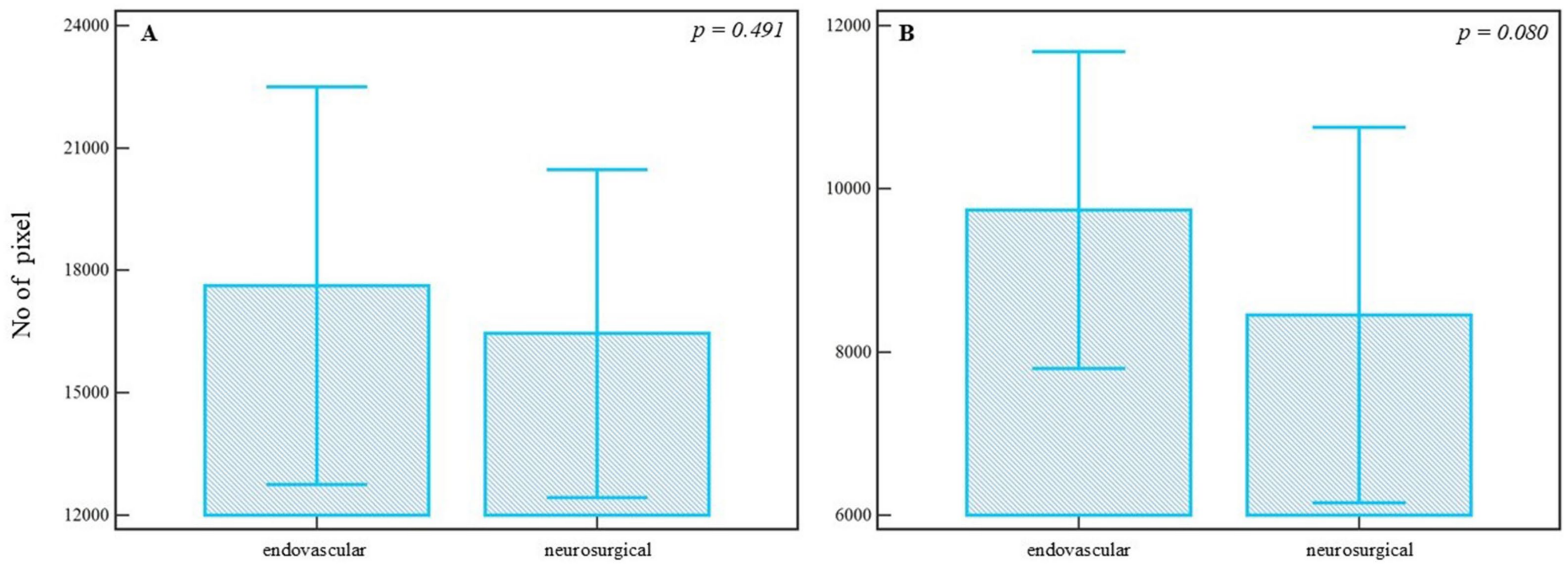
Pixel-based analysis of preoperative and postoperative angiographic images in the neurosurgical treatment group. **(A)** Segmented pixel counts do not differ significantly between pre- and postoperative images (*p* = 0.929, Mann-Whitney *U* test). **(B)** Refined pixel counts also show no statistically significant change (*p* = 0.745, Mann-Whitney *U* test). Bars represent group means; whiskers denote ±1 standard deviation. This plot is not a traditional boxplot but illustrates mean-centered group differences and variability. *p-*values are annotated above the comparisons.

In the neurosurgically treated group (*n* = 30), the analysis of segmented pixels from preoperative and postoperative angiographic images did not demonstrate a statistically significant difference (preoperative median = 13271.000, 95% CI = 11387.649–18083.089; postoperative median = 13352.000, 95% CI = 9998.115–16582.134; *U* = 444.00, *Z* = 0.08, *p* = 0.929). The corresponding effect size was negligible (*r* = −0.08). Similarly, the Wilcoxon Signed-Rank test applied to the segmented pixels did not yield statistical significance (N_pos_ = 17, N_neg_ = 13, *Z* = −0.463, *p* = 0.643), with a small effect size (*r* = −0.08), despite visible individual differences (Figure 4A). Further analysis of refined pixels, accounting for noise reduction and precise segmentation, also showed no statistical significance (preoperative median = 6398.000, 95% CI = 4836.149–8148.228; postoperative median = 6367.500, 95% CI = 5337.444–8215.686; *U* = 428.00, *Z* = 0.325, *p* = 0.745), with Wilcoxon test results confirming no significant change (Npoz = 21, Nneg = 9, *Z* = −1.079, *p* = 0.280), and a small effect size (*r* = −0.20) (Figure 4B).

**Figure 5:**
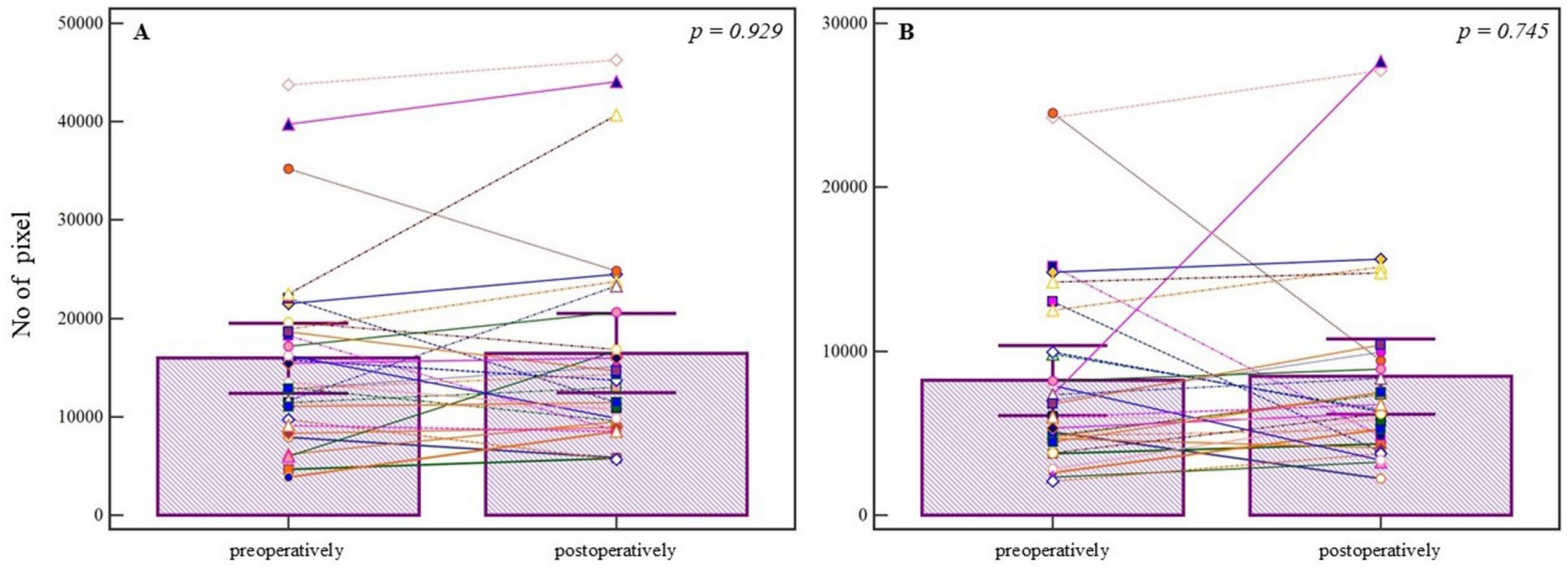
Comparison of postoperative angiographic images between endovascular and neurosurgical treatment groups. **(A)** Segmented pixel counts are higher in the endovascular group, but without statistical significance (*p* = 0.491, Mann-Whitney *U* test). **(B)** Refined pixel counts also show no significant difference between the groups (*p* = 0.080, Mann-Whitney *U* test). Bars represent group means; whiskers denote ±1 standard deviation. This plot is not a traditional boxplot but illustrates mean-centered group differences and variability. *p*-values are annotated above the comparisons.

To assess the differences between treatment approaches, segmented and refined pixel counts from postoperative images were compared. Although the median number of segmented pixels was higher in the endovascular group (median = 15622.000, 95% CI = 10699.135–22484.857) compared to the neurosurgical group (median = 13352.000, 95% CI = 9998.115–16582.134), the difference was not statistically significant (*U* = 279.00, *Z* = 0.689, *p* = 0.491) (Figure 5A), with a small effect size (*r* = 0.10). Similarly, analysis of refined pixels showed no statistically significant difference between groups (endovascular median = 9844.000, 95% CI = 7171.779–12291.201; neurosurgical median = 6367.500, 95% CI = 5337.444–8215.686; *U* = 226.00, *Z* = 1.703, *p* = 0.080) (Figure 5B), though the effect size suggested a trend toward moderate difference (*r* = 0.24). These results suggest that, while numerical differences are evident, the postoperative vascular modifications quantified via pixel-based analysis were not statistically different between endovascular and neurosurgical treatments. Post-treatment intergroup comparisons are summarized in Table 2.

**Table 2:** Post-treatment intergroup comparisons of segmented and refined pixel counts.

Metric	Endovascular (Median [95% CI])	Neurosurgical (Median [95% CI])	*p*-value	Effect size (r)
Segmented pixels	15.622 [10.699–22.485]	13.352 [9.998–16.582]	0.491	0.10
Refined pixels	9.844 [7.172–12.291]	6.368 [5.337–8.216]	0.080	0.24

## Discussion

This study provides one of the first detailed applications of WEKA-based machine learning segmentation in the context of cerebral aneurysm treatment assessment, specifically analyzing vascular changes on pre- and postoperative angiographic images. While prior studies have successfully implemented WEKA in dental and radiographic contexts (25), its application to complex neuroradiological datasets such as DSA remains novel and underexplored. Moreover, we acknowledge that vascular changes are expected following intervention. However, the novelty of our approach lies in leveraging pixel-based image analysis to systematically quantify these changes, even in the absence of direct hemodynamic data. Importantly, while vascular changes are expected following intervention, our method offers a systematic, quantitative way to assess such changes from standard imaging data. Rather than replacing hemodynamic modeling, pixel-based quantification provides an accessible surrogate for morphological remodeling, with potential value as a future adjunctive biomarker for treatment response.

Our results underscore the potential clinical value of pixel-based vascular quantification. In the endovascularly treated group, we identified a statistically significant increase in both segmented and refined vessel pixels post-treatment, suggesting vascular remodeling beyond the immediate site of aneurysm occlusion. Importantly, while vascular changes are expected following intervention, our method offers a systematic, quantitative way to assess such changes from standard imaging data. Rather than replacing hemodynamic modeling, pixel-based quantification provides an accessible surrogate for morphological remodeling, with potential value as a future adjunctive biomarker for treatment response. Prior imaging studies have linked post-intervention vascular geometry changes, such as vessel dilation, curvature, or angular remodeling, to remodeling processes, supporting the plausibility of using segmented surface area as a morphological surrogate in this context (26, 27). This may reflect altered hemodynamics, compensatory vessel dilation, or flow redistribution - phenomena previously linked to endothelial responses and arterial wall adaptation following flow diverter placement (5, 28). It is important to clarify that, in the context of this study, vascular remodeling refers specifically to image-based morphological changes, namely, differences in segmented surface area observed on angiographic images, and does not directly capture functional or histological vessel wall adaptation. Previous studies have reported post-treatment vascular remodeling following flow-diverter placement, often linked to endothelial response and altered hemodynamics, particularly changes in wall shear stress and local flow distribution (5, 28). While our study cannot directly confirm these physiological changes, the observed increase in segmented vessel area may serve as a surrogate imaging marker. Nonetheless, we acknowledge that this finding requires validation through correlation with direct hemodynamic parameters in future studies. In contrast, the neurosurgical group showed no statistically significant change, suggesting that microsurgical clipping may exert a more focal and mechanically stable impact on local vasculature, consistent with previous findings that highlight its durability and limited impact on adjacent vascular territories (6). In addition, while the increase in segmented and refined pixel counts reached statistical significance, the corresponding effect size (Cohen’s d = 0.35) was small. This suggests that the observed morphological changes were modest. In the context of aneurysm follow-up imaging, even subtle post-treatment modifications may carry prognostic value, but further research is needed to determine their true clinical significance.

Clinically, these findings support the utility of machine-learning-enhanced imaging analysis as a powerful adjunct to standard postoperative evaluation. Traditional visual assessment of angiograms often lacks reproducibility and is subject to observer variability. The integration of tools like WEKA allows for quantitative, objective comparisons of vascular states over time, potentially improving decision-making around follow-up imaging, retreatment, or surveillance strategies. Compared to subjective classifications such as the Raymond-Roy occlusion scale, pixel-based segmentation enables continuous, high-resolution quantification of vascular structures, which may be particularly valuable in borderline or ambiguous cases. This approach may complement or even enhance conventional occlusion grading by providing objective, numerical indicators of vascular change that extend beyond simple binary or categorical scales. Importantly, this methodology has the potential to substitute or augment several aspects of current practice: first, by offering automated measurements where radiologists currently rely on visual estimation; second, by detecting subtle hemodynamic changes that may precede clinical or radiographic recurrence; and third, by enabling standardized comparisons in multicenter trials where visual assessment may vary. From a technical standpoint, the segmentation pipeline based on WEKA, Fiji/ImageJ, and Python post-processing provided reliable vessel detection and noise reduction. The apparent discontinuities in segmented images are related to classifier specificity thresholds and projection artifacts, particularly in regions of low contrast or overlapping structures. These were mitigated through post-processing to remove noise and non-connected regions. Quantitative pixel counts were derived from these refined segmentations to ensure consistency. Although not yet fully automated, the ability to segment angiographic datasets with relatively limited training data demonstrates the accessibility and adaptability of this approach across centers. Once refined and validated on larger datasets, this pipeline could be integrated directly into radiology workstations, enabling real-time quantitative feedback during aneurysm follow-up.

Future directions should include the integration of CNNs and semi-supervised learning architectures capable of learning from larger, more heterogeneous datasets. CNNs have already demonstrated superior performance in radiology, particularly in neurovascular applications, enabling pixel-level classification and large-scale pattern recognition with minimal human input (29, 30). These methods offer improved generalizability and reduced manual burden, while AutoML frameworks may further streamline segmentation and post-processing, enabling high-throughput analysis in both research and clinical settings. Importantly, quantitative tools such as this have the potential to enhance reproducibility, scalability, and precision in aneurysm assessment, which are all essential pillars of emerging precision medicine paradigms.

The broader implication is clear: as we move toward precision medicine in neurovascular care, machine learning will play an essential role in transforming how vascular pathologies are detected, quantified, and monitored. This study provides foundational evidence that pixel-based, machine-learning-guided segmentation can detect subtle, treatment-induced vascular changes, offering a reproducible, scalable alternative to subjective image interpretation. As AI continues to be integrated into medical workflows, attention must also be paid to reproducibility, bias mitigation, and validation across diverse clinical populations and institutions (31). While preliminary, these results validate the clinical feasibility of integrating AI-driven tools into routine neuroimaging workflows and reinforce the importance of interdisciplinary collaboration among neurosurgeons, neuroradiologists, and data scientists. It is important to contextualize our findings within the baseline anatomical and clinical differences between groups. The neurosurgical cohort presented with larger and more complex aneurysms on average, including a higher proportion of lesions exceeding 7 mm. These anatomical differences likely contributed to observed variations in clinical course, such as longer hospitalization, and may also influence the extent and pattern of vascular remodeling. While hemodynamic data were not uniformly available, these morphometric observations provide additional interpretive depth and emphasize the importance of future prospective data collection.

As machine learning techniques evolve, future research should focus on developing fully automated, scalable pipelines that incorporate deep learning architectures for real-time, high-throughput angiographic analysis. Such systems could improve reproducibility and reduce observer dependence. Moreover, validation across larger, multicenter and heterogeneous datasets will be essential to ensure robustness and generalizability. Ultimately, pixel-based vascular analysis may contribute to the development of standardized imaging biomarkers to assist in post-treatment surveillance, vascular remodeling assessment, and clinical decision support in neurovascular care.

While this study provides compelling early evidence for the application of machine learning in postoperative aneurysm assessment, several important limitations must be acknowledged to contextualize the findings and guide future research. First, the retrospective and single-center design limits the external validity of the results. Although the inclusion of 60 patients offers a meaningful dataset for exploratory analysis, a larger, multicenter cohort would strengthen statistical power, enhance generalizability across diverse clinical contexts, and reduce the impact of site-specific biases. Additionally, variability in baseline characteristics, such as age, sex, and pre-treatment symptom duration, was not controlled, potentially introducing confounding effects. Second, certain complex aneurysm cases were excluded due to their eligibility for only one treatment modality, which may have skewed the comparative analysis between surgical and endovascular outcomes. This selection bias could affect the observed pixel-based differences in vascular remodeling. One particularly illustrative case involved a patient excluded due to non-adherence to dual antiplatelet therapy, resulting in flow-diverter thrombosis and a fatal outcome. This underscores the clinical importance of post-procedural compliance, an often underestimated but critical factor in long-term treatment success that merits further study. Third, socioeconomic and systemic variables known to influence aneurysm behavior, access to care, and long-term outcomes were not examined. Future studies should aim to integrate these dimensions to better reflect the multifactorial nature of neurovascular disease progression and recovery. Fourth, while all interventions were performed by the same neurosurgeon and interventional radiologist to maintain procedural consistency, this design limits the ability to assess inter-operator variability. In real-world, multi-center environments, operator experience, technique, and protocol adherence can significantly impact procedural outcomes. Fifth, variability in image acquisition, such as differences in contrast injection timing, patient positioning, and the technical parameters of angiographic systems, was minimized in this study by using a single Siemens biplane device under standardized settings. However, the generalizability of the segmentation model across different vendors (e.g., Philips, GE) and acquisition protocols remains untested. Focusing exclusively on MCA aneurysms limits the generalizability of our findings to other aneurysm locations, which may present distinct morphological and imaging characteristics. Cross-platform reproducibility should be a key focus in future validations. Sixth, image quality and signal-to-noise ratio (SNR) were not formally assessed. Although the WEKA classifier was trained on a diverse dataset, its performance under conditions of low contrast or image degradation remains unknown. Evaluating model robustness under suboptimal imaging conditions will be essential for clinical translation. Seventh, and most critically from a technical standpoint, the machine learning pipeline employed in this study was semi-manual and lacked external validation. WEKA’s segmentation framework, although accessible and reproducible, depends heavily on manual classifier training and preprocessing steps such as rotation correction, contrast alignment, and resizing. These operations are labor-intensive and limit scalability. Moreover, the model’s performance was not tested on an independent dataset, leaving its generalizability and overfitting risk unaddressed. In future studies, full automation via deep learning models (e.g., convolutional neural networks) and validation on external, multi-institutional datasets are required to support clinical integration. Additionally, while all angiograms were acquired using standardized parameters, the influence of residual technical variables, such as injection timing, contrast dispersion, and patient positioning, cannot be completely excluded. As such, the observed increase in segmented vessel area may be influenced not only by true vascular remodeling, but also by imaging-related artifacts. Our current pixel-based approach lacks direct validation against physiological hemodynamic indicators, which limits the interpretability of the findings. Importantly, as this methodology evolves, it holds promise for the development of standardized, quantitative imaging biomarkers of vascular remodeling and aneurysm occlusion. The single-center design and limited sample size, particularly in the endovascular group, limit the generalizability of our findings. While procedural standardization was implemented to reduce institutional bias, the sample size may still be underpowered to detect nuanced inter-group differences. Furthermore, although we minimized overfitting by avoiding iterative tuning on the full dataset, small samples inherently constrain the robustness of machine learning outcomes. Future multi-center studies with larger, more heterogeneous populations will be essential for validation. An additional limitation is the potential influence of image preprocessing on pixel counts. Although the same reviewer applied standardized rotation, cropping, and contrast settings for each image pair, manual operations may still introduce minor variability unrelated to biological remodeling. Furthermore, the manual nature of image preprocessing poses potential variability if applied by multiple users. Future work will include consistency checks across reviewers to validate reproducibility. Future studies will explore automated, standardized preprocessing workflows to reduce this source of bias. Future studies will benefit from more standardized and automated pipelines that reduce observer dependence and increase reproducibility. Additionally, the reliance on 2D DSA images without volumetric or hemodynamic reconstruction limits the anatomical and physiological interpretability of observed surface area changes. Additionally, our method simplifies inherently 3D vascular structures into a 2D representation, which may introduce variability related to projection angle and segmentation artifacts. While standardized imaging protocols mitigate some of this variability, future work should include volumetric or cross-sectional imaging to capture remodeling with greater anatomical fidelity.

Despite these constraints, this study serves as a robust proof-of-concept for integrating AI-driven tools into neurovascular imaging workflows. At this stage, the approach should be considered exploratory; however, with proper validation and refinement, it may reach the level of a class IIb clinical decision-support tool, particularly for post-treatment surveillance in endovascular therapy. These findings lay the foundation for future work aimed at scalable, automated analytics to support data-informed aneurysm management.

## Conclusion

This pilot study demonstrates the feasibility of WEKA-based machine learning segmentation for quantifying post-treatment vascular changes in patients with middle cerebral artery aneurysms. In a retrospective cohort of 60 patients, the method detected postoperative increases in segmented and refined pixel counts in a majority of cases, particularly in the endovascular group. These image-based morphological changes may reflect underlying vascular remodeling, although their precise clinical and hemodynamic significance remains to be validated.

While promising, the methodology requires further development to improve reproducibility and clinical applicability. Future work should aim to automate preprocessing steps, incorporate advanced machine learning models, and validate findings across larger, multicenter datasets.

## Data Availability

The raw data supporting the conclusions of this article will be made available by the authors, without undue reservation.
